# Effects of Hexachlorophene, a Chemical Accumulating in Adipose Tissue, on Mouse and Human Mesenchymal Stem Cells

**DOI:** 10.1007/s13770-017-0103-9

**Published:** 2018-01-08

**Authors:** Monika Leśniak, Robert Zdanowski, Milena Suska, Aleksandra Brewczyńska, Wanda Stankiewicz, Małgorzata Kloc, Jacek Z Kubiak, Sławomir Lewicki

**Affiliations:** 1 Departament of Regenerative Medicine and Cell Biology, Military Institute of Hygiene and Epidemiology, Kozielska 4 Street, 01163 Warsaw, Poland; 2 Department of Microwave Safety, Military Institute of Hygiene and Epidemiology, Kozielska 4 Street, 01163 Warsaw, Poland; 3 CNRS, UMR 6290, Institute of Genetics and Development of Rennes, Cell Cycle Group, 35043 Rennes, France; 4 UEB, UMS CNRS 3480, Faculty of Medicine, University Rennes 1, 35043 Rennes, France; 5 The Houston Methodist Research Institute, 6670 Bertner Ave, Houston, TX 77030 USA; 6 Department of Surgery, The Houston Methodist Hospital, 6565 Fannin Street, Houston, TX 77030 USA; 7 MD Anderson Cancer Center, Department of Genetics, The University of Texas, 1515 Holcombe Blvd, Houston, TX 77030 USA

**Keywords:** Hexachlorophene, Mesenchymal stem cells, Adipose tissue, Unbilical cord, Apoptosis

## Abstract

The hexachlorophene (HCP) is a highly lipophilic chlorinated bisphenol present in hygienic and dermatological products. The HCP accumulates preferentially in adipose tissue that is a privileged source of mesenchymal stem cells (MSCs). The evaluation of the potential effects of HCP on MSCs is important for their medical application. Here we examined the effects of HCP on murine adipose tissue-derived stem cells (ADSCs) and human umbilical cord-derived stem cells (UCSCs) in cell culture. We found that 10^−4^ and 10^−5^ M HCP inhibits proliferation, osteogenesis and increases apoptosis of ADSCs and UCSCs. While the effect of HCP on proliferation and differentiation potential of these two cell lines was similar, the UCSCs appeared much more resistant to HCP-induced apoptosis than ADSCs. These results suggest that the adipose tissue-derived ADSCs have higher sensitive for HCP than umbilical cord-derived UCSCs and indicate that the umbilical cord can be a preferable source of MSCs for prospective medical applications in the future.

## Introduction

The ability of pluripotent stem cells to differentiate into osteoblasts, chondrocytes, myoblasts, adipocytes and neurons makes them irreplaceable tool in regenerative medicine [1, 2]. The stem cells isolated from adult human tissue contain a high percentage of Mesenchymal Stem Cells (MSCs). Studies performed in recent years showed that the adipose (fat) tissue might be a preferred source of MSCs due to its availability and ease of acquisition. However, the adipose tissue has affinity for and accumulates highly lipophilic chemicals, such as insecticides, polychlorinated biphenyls, hexachlorophene, polycyclic aromatic hydrocarbons, which are present in food (especially produced in industrialized farms) and personal hygiene products [3]. These chemicals constitute a potential human health risk; a sudden loss of fat tissue after intense exercise or extreme weight loss causes rapid release of adipose tissue-accumulated chemicals into the blood, liver, kidneys and brain. In addition, these chemicals may affect proliferation and/or differentiation of adipose tissue-derived MSCs.

Hexachlorophene [2,2'-methylenebis-(3,4,6-trichlorophenol)] (HCP) is a chlorinated bisphenol with strong bacteriostatic properties against many Gram-positive bacteria (including *Staphylococcus*) [4] that is used in soaps, creams and dermatological preparations. Because HCP is very resistant to biotransformation it accumulates in the environment and bioaccumulates in food chain [5]. Animal studies have shown a clear chronic negative effect of HPC on human health. HCP administered orally or vaginally in high doses was embryotoxic and teratogenic in rats [6]. Lokanatha et al. [7] showed that HCP had, through the inhibition of succinate dehydrogenase, neurotoxic effects on rat brain. The poisoning symptoms of HCP are neurologic, cardiovascular, respiratory, gastrointenstinal and dermatological. The range of lethal dose in adult human is 2–10 g per kg of body mass [8]. However, the minimal lethal dose is unknown. Because of its high hydrophobicity the HCP rapidly accumulates in adipose tissues and central nervous system (CNS). Within the nervous system the myelin has a significant affinity for HCP. Experimental studies showed inhibitory effect of HCP on synthesis of myelin possibly by inhibition of oxidative phosphorylation [5]. HCP is metabolized in the liver by glucuronidation and is excreted into bile.

Here we investigated the effect of various concentrations of HCP on the proliferatation, apoptosis and differentiation into osteaoblasts of MSCs from two different sources: adipose tissue and umbilical cord. We also tested whether preincubation of MSCs with HCP affects their differentiation into osteoblasts.

## Materials and methods

### Animals and human tissues samples

Fat tissue was isolated form 8 weeks C57BL/6 mice. Mice were sacrificed by anesthetic overdose (pentobarbital, 400 mg/kg; Polypharm S.A., Warsaw, Poland). Fat tissue was isolated from gonadal and kidney localization under aseptic conditions (laminar flow cabinet) and transferred to falcon probe which contained 10 ml PBS with antibiotics penicillin–streptomycin (500 IU/ml, Thermo Fisher Scientific, Poland) until use.

Human umbilical cord samples were downloaded postpartum. After isolation umbilical cord samples were transferred to falcon probe which contained 30 ml PBS with antibiotics (penicillin–streptomycin (500 IU/ml, Thermo Fisher Scientific, Poland) until use.

### Cell isolation and culture

Experiments were performed on two primary cells lines: adipose derived stem cells (ADSCs)—mesenchymal stem cells isolated from adipose tissue of C57BL/6 mice, and umbilical cord stem cells (UCSCs)—mesenchymal stem cells isolated from human umbilical cord.

ADSCs were isolated by the method described by Gronthos et al. [9] with our own modification. The adipose tissue was digested in type 1 collagenase (1 mg/ml; Sigma Aldrich, Poland) for 1 h at 37 °C with shaking in water bath. Digested tissue was strained through 40 μm strainer, washed with culture medium (DMEM F12, 10% FBS, penicillin/streptomycin, Thermo Fisher Scientific, Poland) and centrifuged for 5 min at 500×*g*. The cells recovered in pellets were counted and seeded onto 6 well dishes at 1.5 × 10^6^ cells/well and cultured at 37 °C in 5% CO_2_. The cell culture medium was changed after 24 h. Cells were grown to 90% confluency, washed with PBS, harvested by trypsinization (0.25% trypsin in PBS) and seeded into 75 cm^2^ culture bottles at 10^6^ cells/bottle. Cells were cultured up to 18 passage. The proliferation time between passages equals 96 h.

Human UCSCs were isolated from umbilical cord Wharton’s jelly by the method described by Ishige et al. [10] with our own modification. Fragments of umbilical cord were cut into small pieces and cultured at 37 °C in 5% CO_2_ in DMEM medium containing 4.5 g/l of glucose (Thermo Fisher Scientific, Poland), 20% FBS (Thermo Fisher Scientific, Poland) and penicillin/streptomycin (Thermo Fisher Scientific, Poland). The culture medium was replaced once a week to achieve 90% cell confluency. Cells were rinsed twice with PBS (Thermo Fisher Scientific, Poland), trypsinized (0.25% trypsin in PBS, Thermo Fisher Scientific, Poland) and seeded in 25 cm^2^ cell culture flasks at 3 × 10^5^ cells/flask. Cells were cultured up to 24 passage. The proliferation time between passages equals 72 h.

After 2nd and 3rd passage, ADSCs and UCSCs cells were frozen in culture medium with 10% DMSO (Sigma Aldrich, Poland) and stored under liquid nitrogen.

Mesenchymal stem cells isolated from mouse adipose tissue and human umbilical cord were derived from tissue lipophilic compounds free.

### Flow cytometry analysis

The ADSCs and UCSCs were collected using Acutase (BD Biosciences, Poland) and washed in PBS (Sigma Aldrich, Poland). The presence of surface markers: CD29^+^, CD105^+^, CD106^+^ and CD45^−^ (Becton–Dickinson, Poland) was determined in 2nd and 3rd passage cells using flow cytometer FACS Calibur and the results were analyzed by Cell Quest pro software (both from BD, USA).

### Hexachlorophene preparation

HCP was purchased from Sigma Aldrich, Poland. HCP was dissolved in 10^−1^ M DMSO (Sigma Aldrich, Poland), aliquoted and stored in glass bottles in the dark at 4 °C. We have used 10^−7^–10^−4^ M HCP concentration in the culture medium. The DMSO content in the samples was 0.01%.

### Proliferation assays

#### Cell culture

ADSCs or UCSCs in a log phase stage of growth were harvested by trypsinization (0.05% solution, Thermo Fisher Scientific, Poland) and pelleting at 300×*g* for 5 min. Cell pellets were resuspended in culture medium and 1.5 × 10^4^ cells/ml were seeded into 96-well plate (200 µl per well). After 24 h cells were washed twice with PBS, and 10^−7^–10^−4^ M HCP was added in fresh culture medium. Control cells were grown in culture medium without HCP and with 0.01% DMSO. The proliferation assay will be evaluated using three independent cell number comparison tests which were performed as previously described [11].

#### MTT assay

24, 48 and 72 h after HCP addition the culture medium was discarded and fresh culture medium containing MTT (1 mg/ml, Sigma Aldrich, Poland) was added directly to the wells. After 2 h incubation (37 °C, 5% CO_2_,) the medium was discarded, cells were rinsed 3 times with PBS and to dissolve the formazan crystals the 100 µl of DMSO was added to the wells. The absorbance was measured at 570 nm using FLUOstar Omega reader (BMG Labtech, Sweden).

#### Neutral Red assay

24, 48 and 72 h after HCP addition the culture medium was discarded and fresh culture medium containing Neutral Red (25 mg/ml; Sigma Aldrich, Poland) was added directly to the wells. After 2 h of incubation (37 °C, 5% CO_2_,) the medium was discarded and cells were rinsed 3 times with PBS. Subsequently, in order to dissolve the pigment, cells were rinsed with 100 µl of 1% acetic acid, 50% ethanol, 49% water solution (Sigma Aldrich). The absorbance was measured at 570 nm using FLUOstar Omega reader (BMG Labtech, Sweden).

#### Sulforodamine B assay

24, 48 and 72 h after HCP addition the culture medium was discarded and cells were fixed with 50% trichloroacetic acid (Sigma Aldrich, Poland,; 100 μl per well, 1 h, 4 °C). Fixed cells were washed 3 times in water, air dried and 100 µl of sulforodamine B (0.4% in 1% acetic acid; Sigma Aldrich, Poland) was added. After 30 min incubation cells were washed with 1% acetic acid 4 times, air dried and 10 mM Trisma^®^base (100 µl/well; Sigma Aldrich, Poland) was added to dissolve the pigment. The fluorescence was measured at excitation 570 nm (± 10) and emission 590 nm (± 10) using FLUOstar Omega reader (BMG Labtech, Sweden).

### Apoptosis assay

ADSCs or UCSCs in a log phase growth were harvested and seeded at 3 × 10^4^ cells/well and 5x10^4^ cells/well, respectively, in 24-well plate (1 ml/well). 24 h later cells were incubated with 10^−7^–10^−4^ M HCP. Control cells were grown in culture medium without HCP and with 0.01% DMSO. After 24 and 72 h incubation cells were washed twice with PBS and pelleted (500×*g*, 5 min). Cell pellets were washed twice with PBS, centrifuged and resuspended in annexin binding buffer with addition of 4.5 µl annexin V-FITC antibody (eBioscence, Poland) and 8 µl propidium iodide 1 mg/ml (Sigma Aldrich, Poland). After 20 min incubation cells were washed with PBS and analyzed by flow cytometry (FACS Calibur, BD, USA). Evaluation of live, apoptotic and necrotic cells was performed using Cell Quest software.

### Osteogenesis of ADSCs and UCSCs

#### ADSCs and UCSCs osteogenesis in the presence of HCP in osteogenic induction medium

ADSCs or UCSCs in a log phase were plated in 24-well plate at 4 × 10^4^ cells/well (ADSCs) and 2 × 10^4^ cells/well (UCSCs). Cells were grown to 95% confluency and osteogenic induction medium (DMEM 4.5 g/l d-Glucose or DMEM F12, 10% FBS, 200 µM l—ascorbic acid 2-phosphate, 100 nM dexamethasone, 10 mM β-Glycerophosphate—all from Sigma Aldrich, Poland) was added directly to the wells. The composition of the osteogenic induction medium was described by Kim et al. [12] and Wang et al. [13] and the concentration of the individual compounds were selected experimentally by us. The HCP was added at 10^−7^–10^−4^ M. The control cells were cultured in osteogenic induction medium without HCP and with 0.01% DMSO (positive control) or culture medium without osteogenic supplement, HCP and with 0.01% DMSO (negative control). Cells were cultured for 3 weeks and medium was changed every 3–4 days.

#### ADSCs and UCSCs preincubation with HCP followed by osteogenesis without HCP in osteogenic induction medium

Mesenchymal Stem Cells in a log phase stage of growth were plated in 25 cm^2^ flasks at density 4 × 10^5^ cells/flask (ADSCs) and 2 × 10^5^ cells/flask (UCSCs). 24 h later cells were treated with different concentrations (10^−7^–10^−4^ M) of HCP. Cells were grown to 90-95% confluence, harvested by trypsinization and seeded in 24-well plate at 4 × 10^4^ cells/well (ADSCs) and 2 × 10^4^ cells/well (UCSCs). After cells had formed monoleyer they were cultured for 3 weeks in osteogenic induction medium without HCP. Medium was changed every 3–4 days. The positive control cells were cultured in osteogenic induction medium without HCP and with 0.01% DMSO. The negative control cells were cultured in medium without osteogenic supplements and with 0.01% DMSO.

#### Alizarin red staining

After 3 weeks of osteogenic differentiation cells were fixed in 4% paraformaldehyde (Sigma Aldrich, Poland) for 20 min. Fixed cells were washed with PBS and stained with Alizarin Red S (2 g/100 ml of distilled water; Sigma Aldrich, Poland) pH 4.1–4.3 for 20 min.

Alizarin Red S stains red the calcium deposits formed in the differentiated cells (osteoblasts) The results are presented using a five-point visual scale: undifferentiated cells (−), differentiated cells depending on the intensity of staining (+), (++), (+++), (++++).

### Statistical analysis

Statistical analysis was performed using unpaired t-tests and one-or two-way analysis of the variance, followed by the Tukey test or Bonferroni correction (in the case of a normal distribution) or non-parametric Kruskal–Wallis and Mann–Whitney *U* tests (in the case of abnormal distribution). Assessment of the distribution of data was evaluated using the Shapiro–Wilk test. GraphPad Prism software was used (ver. 5; GraphPad Software, Inc., La Jolla, CA, USA). *p* < 0.05 was a statistically significant difference.

## Results

### Mesenchymal cells phenotype

**Fig. 1:**
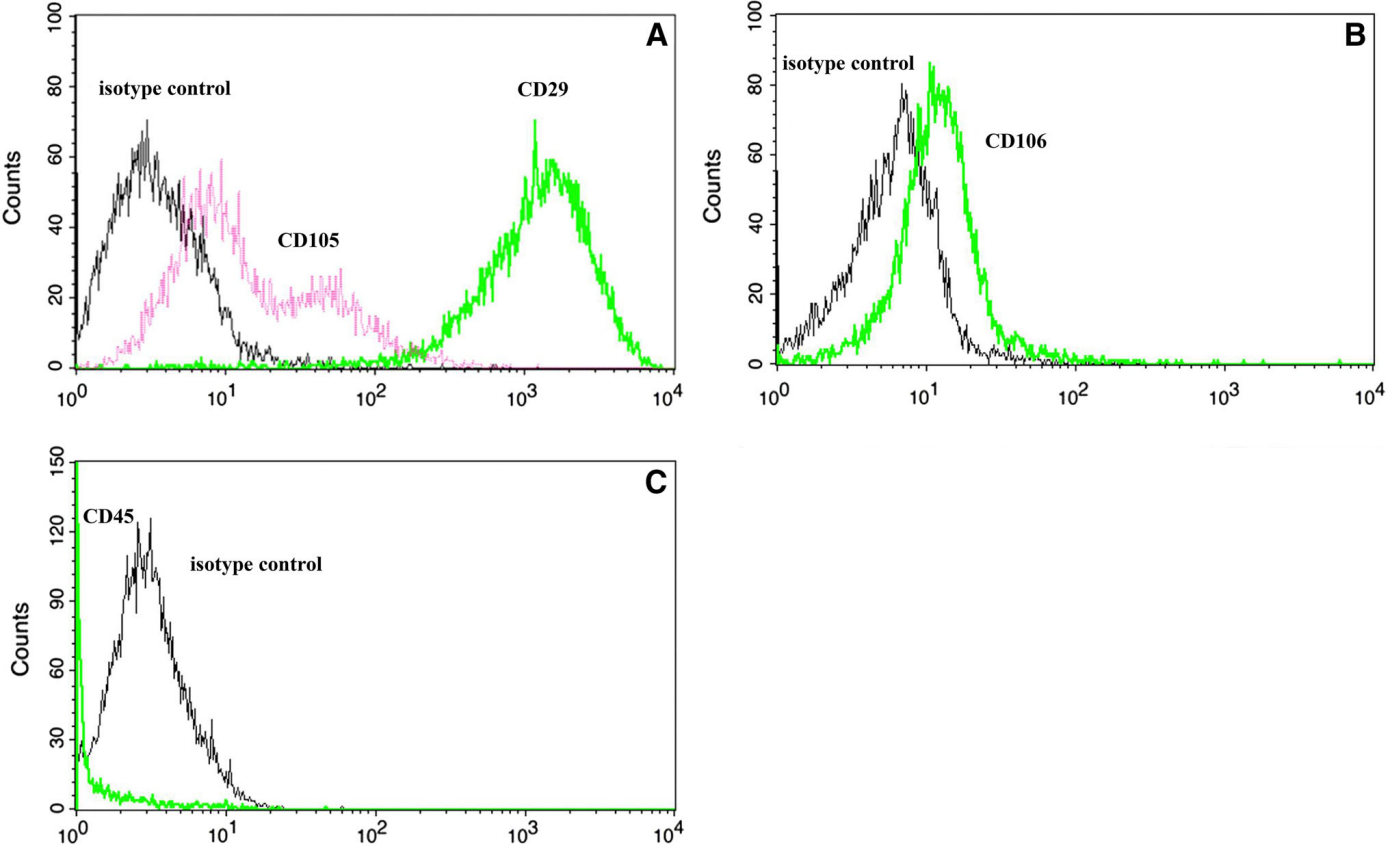
Stem cell surface markers expressed by ADSCs **A** CD 105, CD 29, **B** CD 106 and **C** CD 45

Mice adipose-derived stem cells (ADSCs), after third passage of *in vitro* culture, expressed CD29, CD105 and CD106 surface markers. Over 95% of cells expressed CD29, CD105 and CD106 markers. The highest expression was found for CD29 marker. There was a weak expression of CD105 and CD106 and the CD45 positive cells were absent. The results are presented in Fig. 1.

**Fig. 2:**
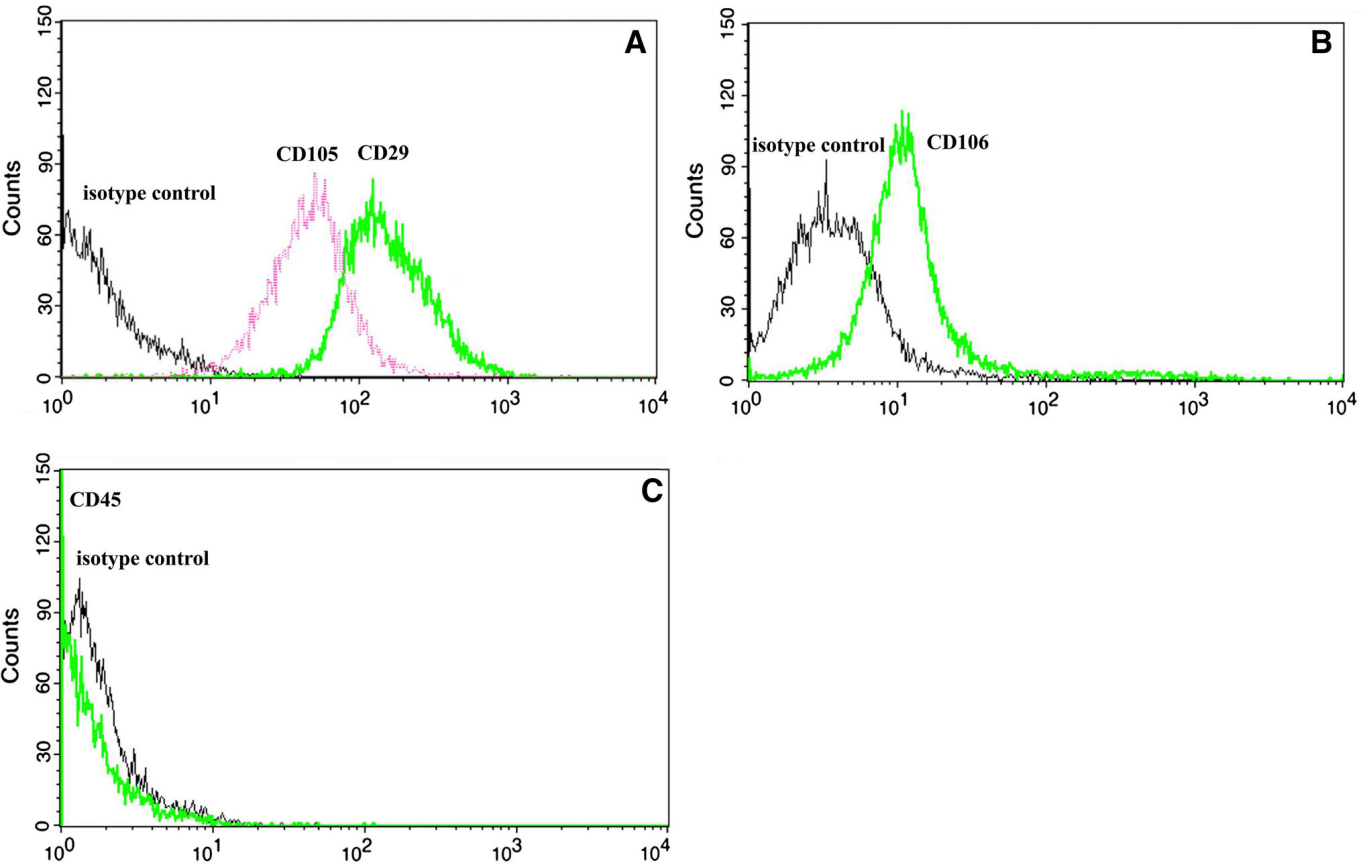
Stem cells surface markers expressed by UCSCs **A** CD 105, CD 29, **B** CD 106 and **C** CD 45

Human UCSCs also expressed CD29, CD105 and CD106 stem cell surface markers Over 95% of cells expressed CD29, CD105 and CD106 markers. The CD45 positive cells were absent. The highest level of expression was observed for CD29 > CD105 > CD106. The UCSCs also expressed CD73, CD90 surface markers (data not shown) and were negative for CD34 expression (data not shown). The results are presented in Fig. 2.

### Proliferation assays

#### ADSCs

**Fig. 3:**
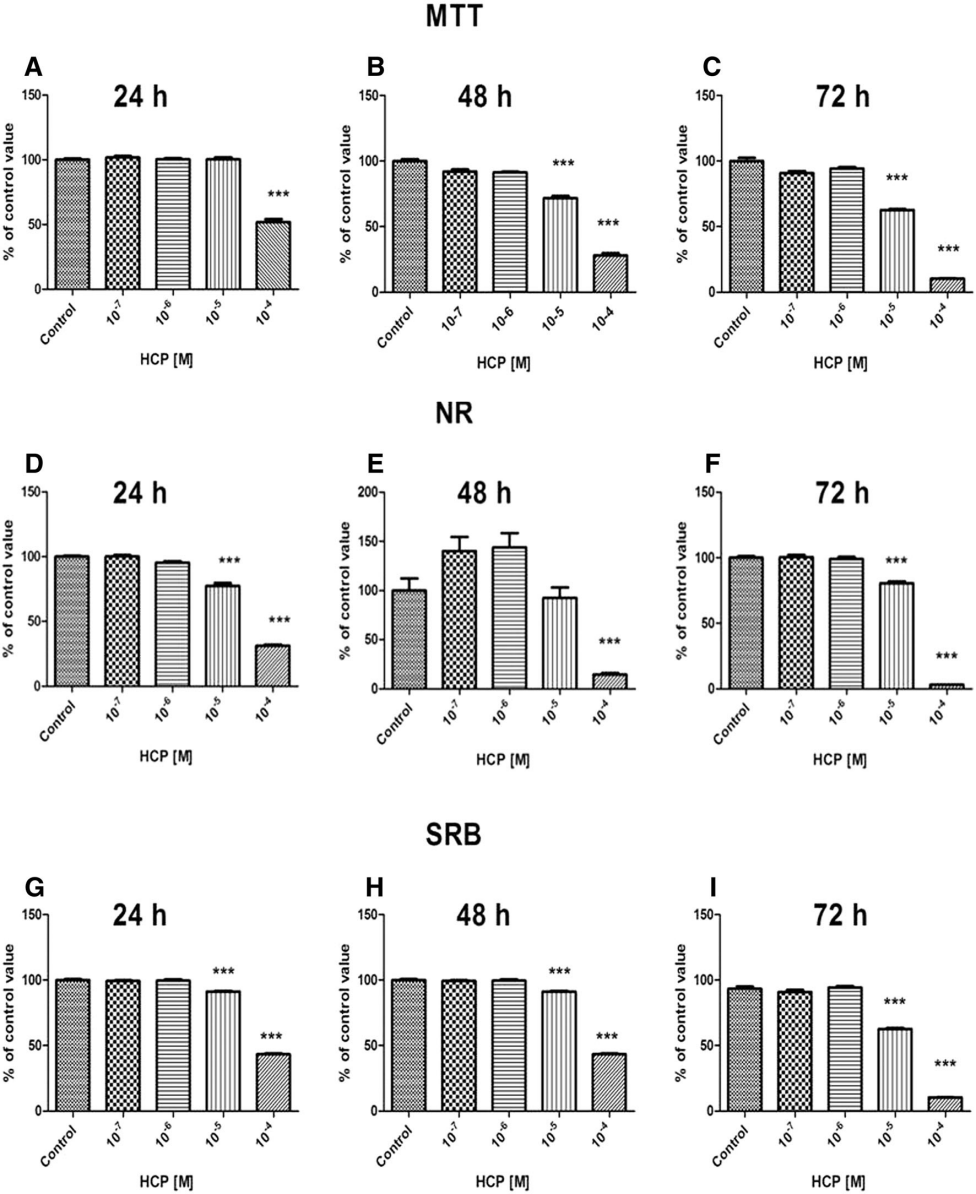
Effect of HCP treatment on ADSCs proliferation assessed by MTT assay **A** 24 h, **B** 48 h, **C** 72 h; NR assay **D** 24 h, **E** 48 h, **F** 72 h and SRB assay, **G** 24 h, **H** 48 h, **I** 72 h (mean ± SEM)

The results of MTT, NR and SRB, three independent cell number comparison tests, showed that there was no significant change in proliferation of ADSCs after 24–72 h treatment with 10^−7^ and 10^−6^ M HCP in comparison to the control. The higher concentration of HCP (10^−5^ and 10^−4^ M) significantly decreased (75% of the control value, *p* < 0.0001) cell proliferation after 72 h. Toxic effect of the highest concentration (10^−4^ M) of HCP was already visible after 24 h of treatment. Figure 3 shows the results (presented as a mean ± SEM) from 3 independent experiments.

#### UCSCs

**Fig. 4:**
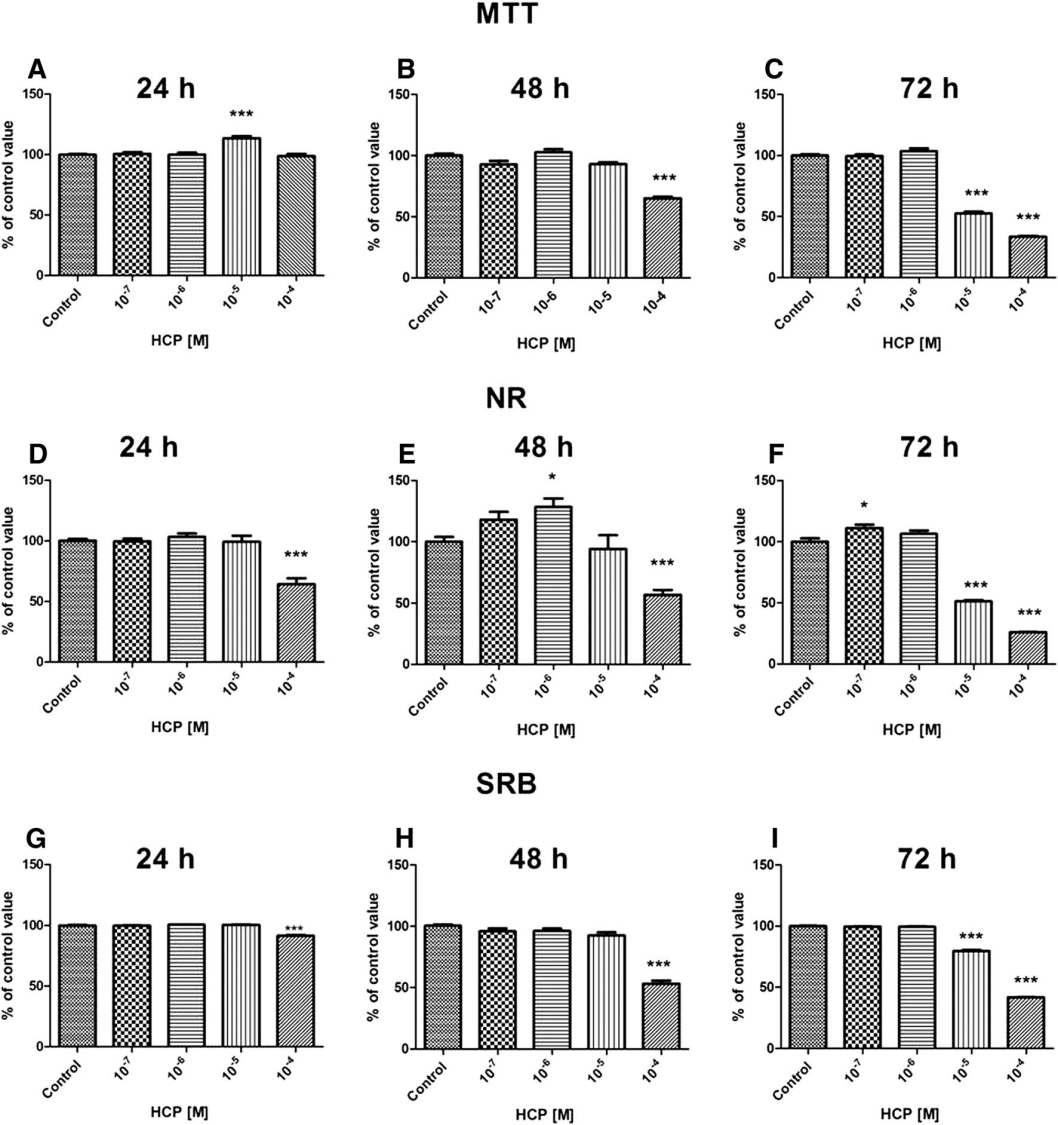
Effect of HCP treatment on UCSCs proliferation assessed by MTT assay **A** 24 h, **B** 48 h, **C** 72 h; NR assay **D** 24 h, **E** 48 h, **F** 72 h and SRB assay, **G** 24 h, **H** 48 h, **I** 72 h (mean ± SEM)

The results of MTT, NR and SRB proliferation assays showed that there was no significant change in proliferation of UCSCs after 24-72 h treatment with 10^−7^ and 10^−6^ M HCP in comparison to the control. There was slight, but statistically significant increase of proliferation after 48 h treatment with 10^−6^ M HCP (*p* < 0.05) and 72 h treatment with 10^−7^ M HCP (*p* < 0.05) when compared to the control. The highest concentration of HCP (10^−4^ M) caused significant reduction of cell proliferation, especially after 72 h treatment (Fig. 4). The results from 3 independent experiments are shown as a mean ± SEM.

### Apoptosis assay

#### ADSCs

**Fig. 5:**
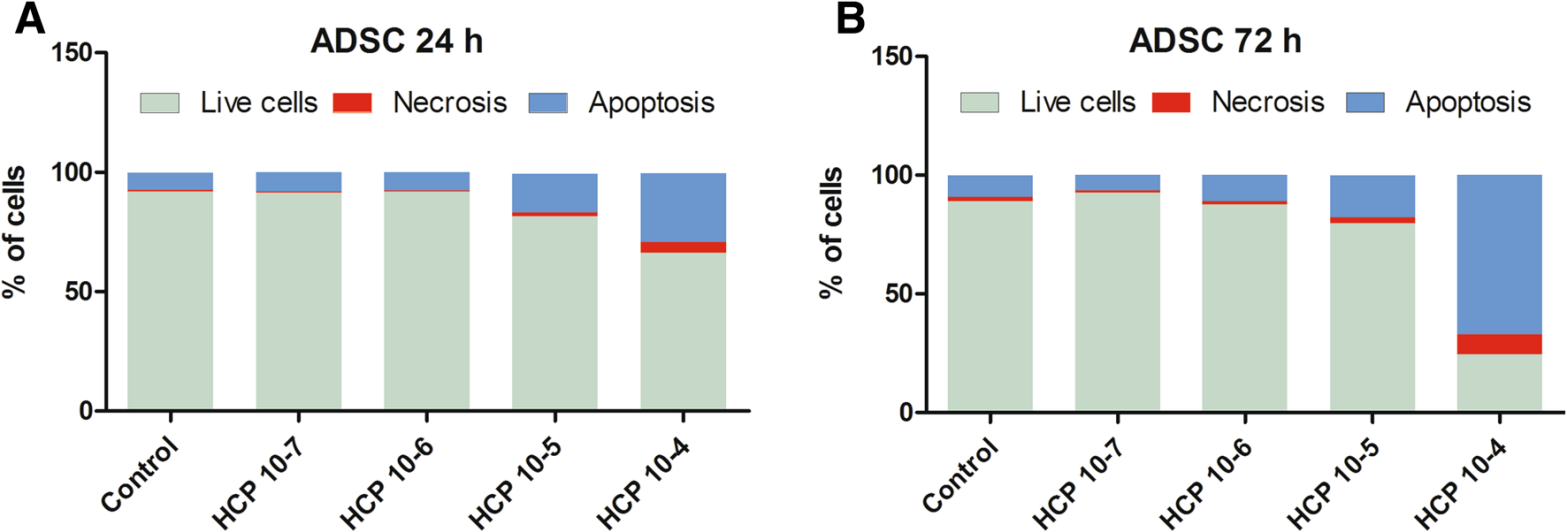
**A**, **B** Effect of HCP on ADSCs apoptosis and necrosis after 24 and 72 h treatment

The treatment (24 and 72 h) with HCP (10^−7^ and 10^−6^ M) did not affect the number of necrotic or apoptotic ADSC cells when compared to the control (Fig. 5A, B). However, there was statistically significant dose-dependent decrease in the percentage of live cells after treatment with 10^−5^ and 10^−4^ M HCP. HCP in concentration 10^−5^ and 10^−4^ M caused significant increase in necrosis and especially in apoptosis. The highest number of necrotic and apoptotic cells were found in 10^−4^ M HCP after 72 h treatment (*p* < 0.0001) (Fig. 5B). The results from 3 independent experiments are shown as a mean.

#### UCSCs

**Fig. 6:**
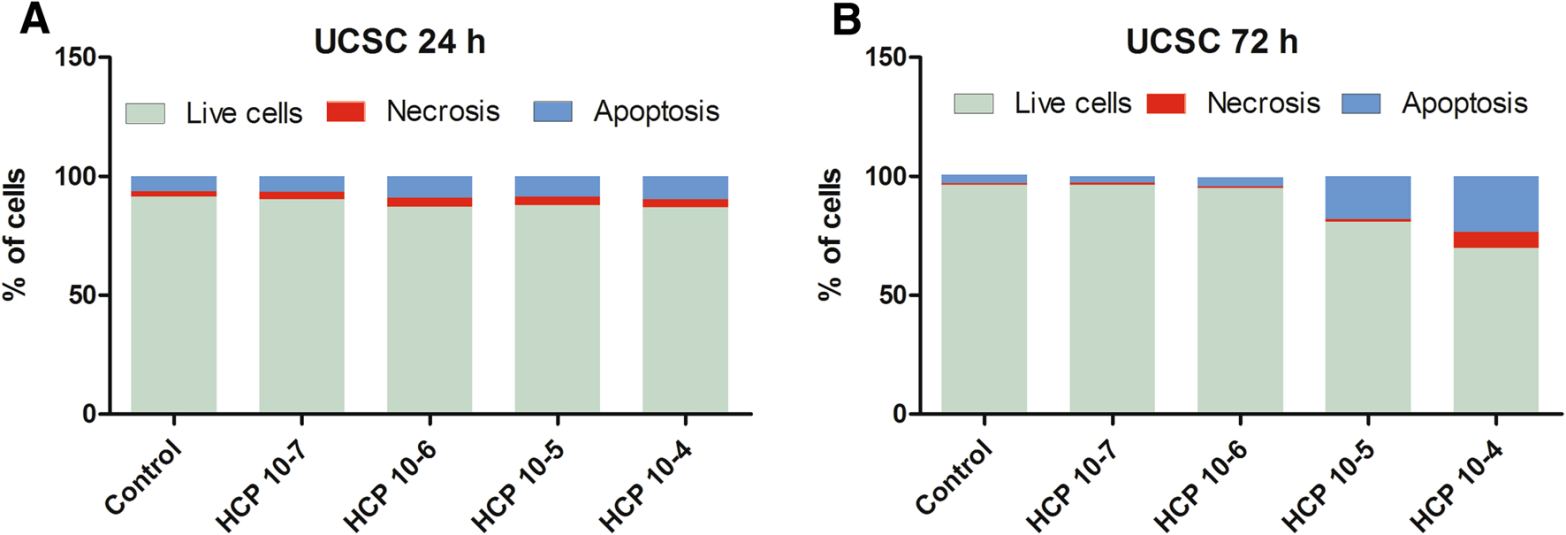
**A**, **B** Effect of HCP on UCSCs apoptosis and necrosis after A 24 and B 72 h treatment

Similar to the results obtained for ADSCs, the 10^−7^ and 10^−6^ M HCP did not affect the percentage of live UCSCs when compared to the control group (Fig. 6A, B). However, the UCSCs were less susceptible to HCP treatment than ADSCs. After 24 h HPC treatment in concentration form 10^−7^ to 10^−4^ M there was no significant change in the percentage of viable cells or in the percentage of necrotic or apoptotic cells. After 72 h HCP treatment in concentration of 10^−5^ and 10^−4^ M there was statistically significant dose depended decrease in the percentage of viable cells (about 17%, *p* < 0.0001, and about 28% *p* < 0.001, respectively). In concentration of 10^−5^ M HCP the cells mainly died by apoptosis and there was no significant increase in necrosis. The highest concentration of HCP (10^−4^ M) caused increase in both necrosis (about 930%, *p* < 0.001) and apoptosis (about 630%, *p* < 0.001) (Fig. 6B). The results from 3 independent experiments are shown as mean values.

### Osteogenesis of ADSCs and UCSCs

#### ADSCs and UCSCs osteogenesis in the presence of HCP in osteogenic induction medium

**Fig. 7:**
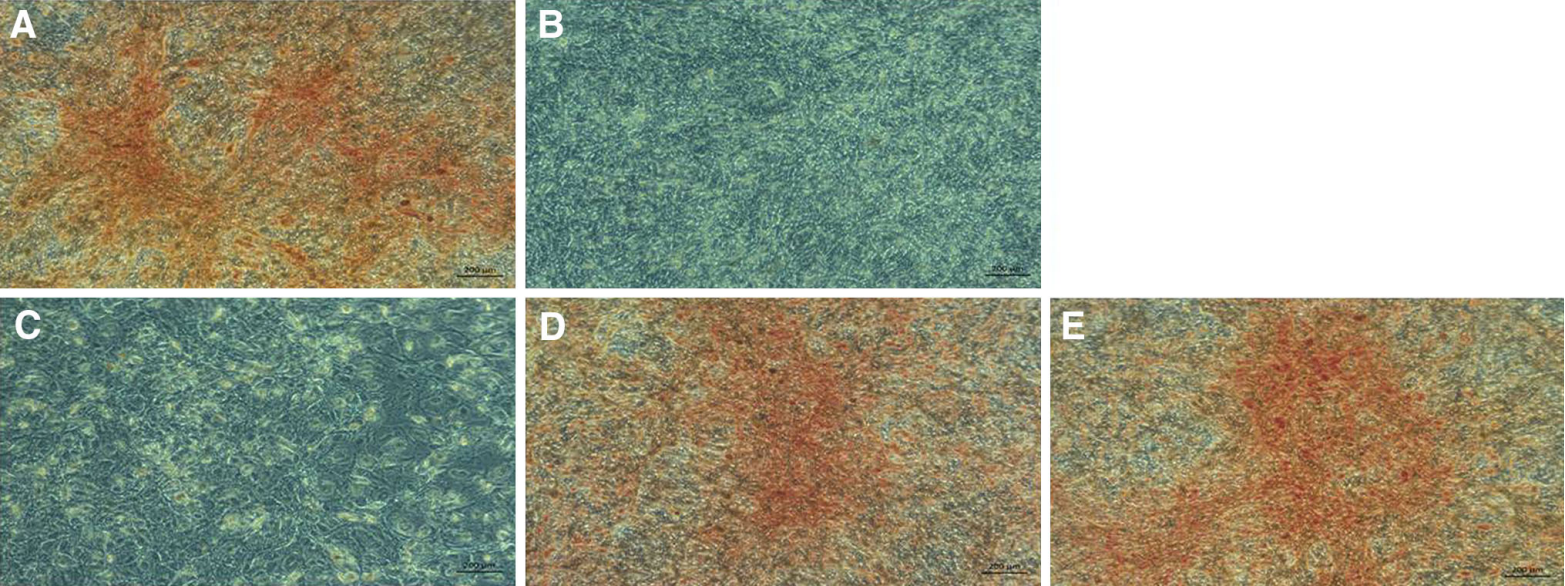
Alizarin Red staining on 21th day of ADSCs osteogenesis (calcium deposits formed in the differentiated cells (osteoblasts) are orange/red color). **A** Positive control (differentiated cells without HCP). **B** Negative control (cells cultured in culture medium without osteogenesis factors). **C** HCP 10^−5^ M, **D** HCP 10^−6^ M, **E** HCP 10^−7^ M. Images acquired by inverted light microscope (ZEISS AX10, Poland; magnification ×100)

**Fig. 8:**
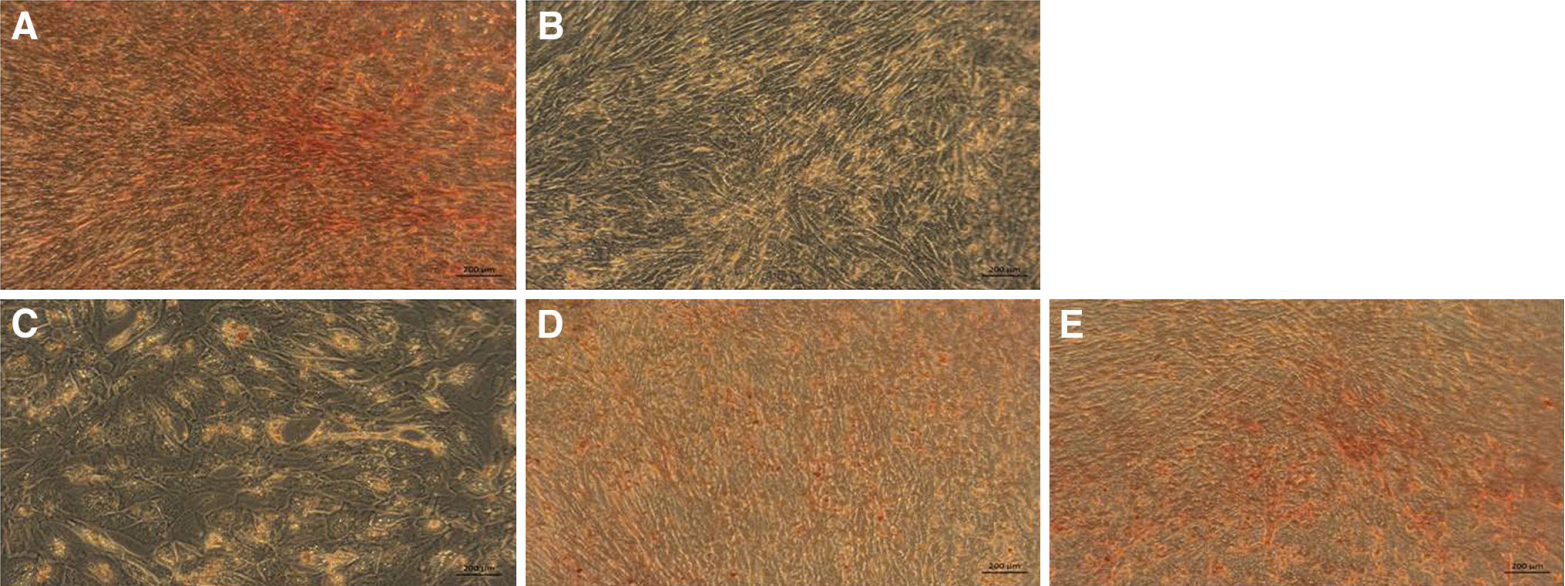
Alizarin Red staining on 21th day of UCSCs’ osteogenesis (calcium deposits formed in the differentiated cells (osteoblasts) are orange/red color). **A** Positive control (differentiated cells without HCP). **B** Negative control (cells cultured in culture medium without ostegenesis factors). **C** HCP 10^−5^ M, **D** HCP 10^−6^ M, **E** HCP 10^−7^ M. Images acquired by inverted light microscope (ZEISS AX10, Poland; magnification ×100)

The 10^−4^ M HCP added to the osteogenic induction medium caused death of both ADSCs and UCSCs cells before the end of experiment (not shown). The addition of 10^−5^ M HCP significantly inhibited osteogenesis of ADSCs and UCSCs (there was no visible calcium deposits after Alizarin Red staining Figs. 7C, 8C). Similar effect was observed for negative control (Figs. 7B, 8B). Treatment with lower concentration (10^−6^ and 10^−7^ M) of HCP resulted in lower differentiation level (Figs. 7D–E, 8D–E) when compared to the control (Figs. 7A, 8A). Results of microscopic evaluation of the intensity of Alizarin Red staining are shown in Table 1.

**Table 1:** ADSCs and UCSCs Alizarin Red staining intensity (microscopic evaluation)

Stem cells	Control positive	Control negative	HCP		
			10^−5^ M	10^−6^ M	10^−7^ M
ADSCs	++++	–	–	++	++
UCSCs	++++	–	–	++	++

#### ADSCs and UCSCs preincubation with HCP followed by osteogenesis without HCP in osteogenic induction medium

**Fig. 9:**
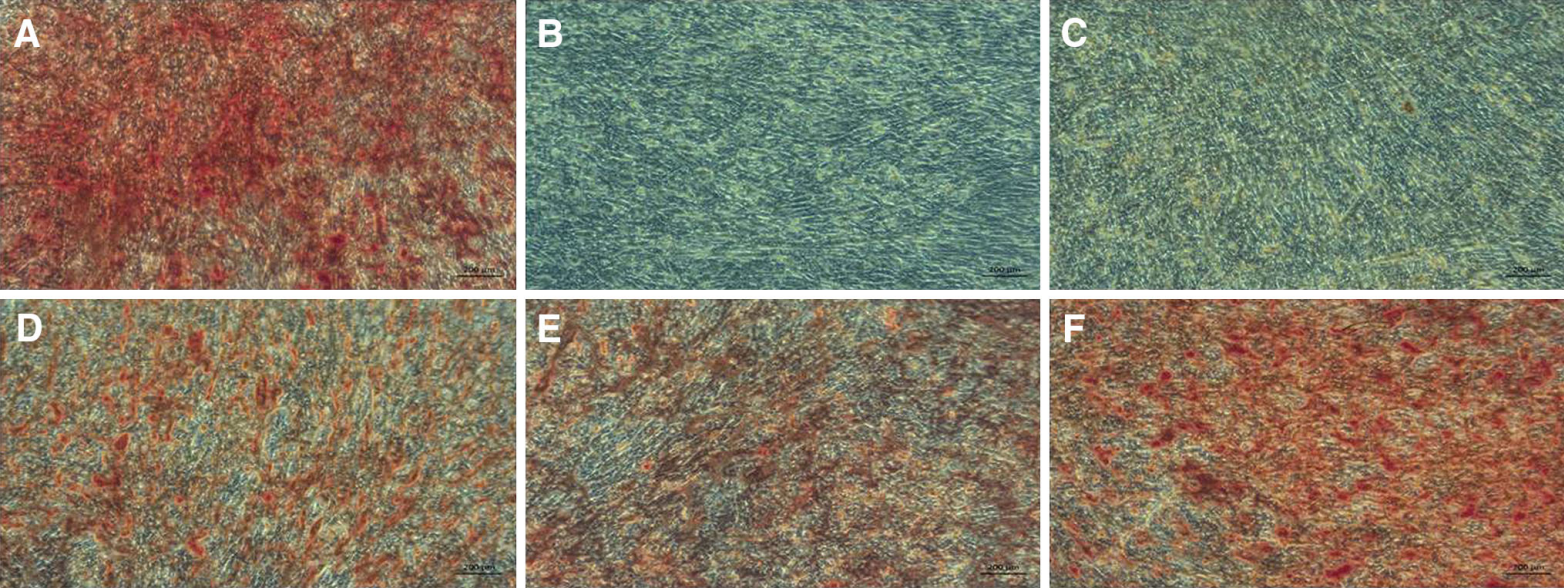
Alizarin Red staining on 21th day of ADSCs osteogenesis (calcium deposits formed in the differentiated cells (osteoblasts) are red–orange/red color). **A** Positive control (differentiated cells without preincubation with HCP in osteogenic induction medium). **B** Negative control (cells cultured in culture medium without preincubation with HCP). **C**–**E** Differentiated cells preincubated with HCP before the beginning of the differentiation process. **C** 10^−4^ M HCP. **D** 10^−5^ M HCP. **E** 10^−6^ M HCP. **F** 10^−7^ M HCP. Images acquired by inverted light microscope (ZEISS AX10, Poland; magnification ×100)

**Fig. 10:**
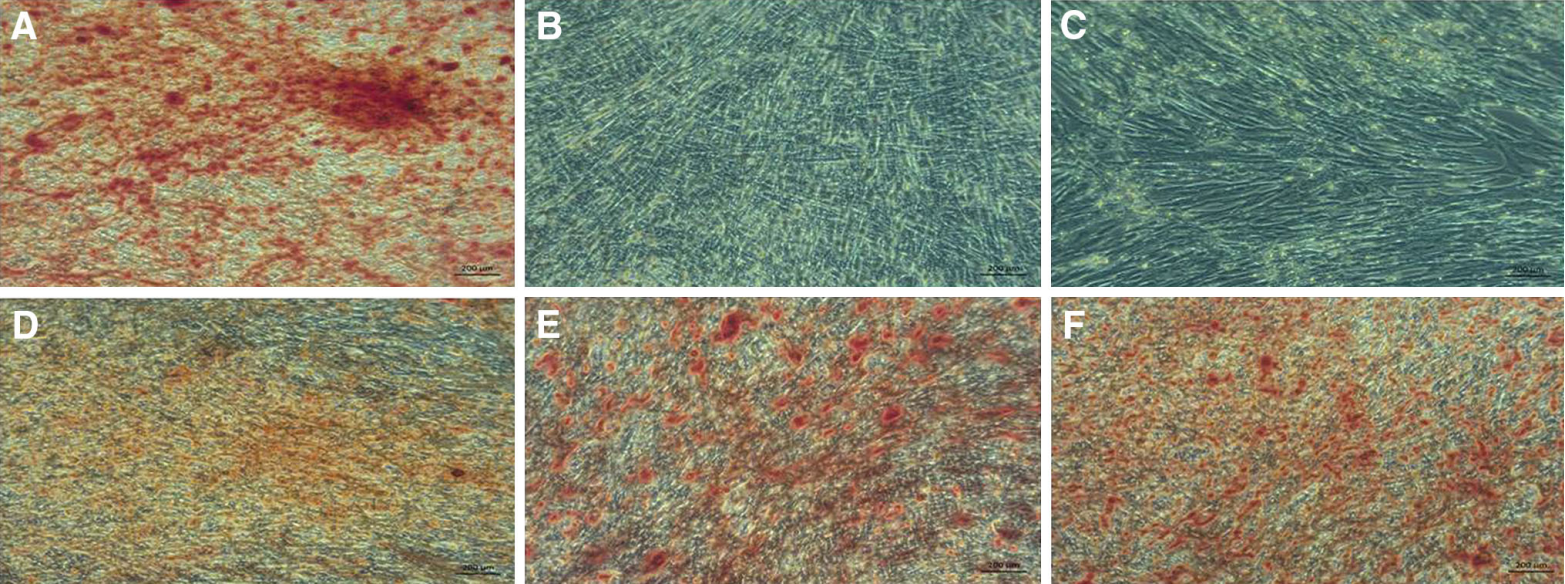
Alizarin Red staining on 21th day of UCSCs osteogenesis (calcium deposits formed in the differentiated cells (osteoblasts) are orange/red color). **A** Positive control (differentiated cells without preincubation with HCP in osteogenic induction medium). **B** Negative control (cells cultured in culture medium without preincubation with HCP). **C**–**E** Differentiated cells preincubated with HCP before the beginning of the differentiation process. **C** 10^−4^ M HCP. **D** 10^−5^ M HCP. **E** 10^−6^ M HCP. **F** 10^−7^ M HCP. Images acquired by inverted light microscope (ZEISS AX10, Poland; magnification ×100)

ADSCs and UCSC cells preincubated with 10^−4^ M HCP remained undifferentiated (Figs. 9C, 10C) similar to negative control (Figs. 9B, 10B). The preincubation with HCP in lower concentration (10^−5^–10^−7^ M) did not inhibit osteogenesis (Figs. 9D–F, 10D–F) but the level of differentiation was lower than in positive control (Figs. 9A, 10A). Results of the microscopic evaluation of the intensity of Alizarin Red staining are shown in Table 2.

**Table 2:** ADSCs and UCSCs Alizarin Red staining intensity (microscopic evaluation)

Stem cells	Control positive	Control negative	HCP			
			10^−4^ M	10^−5^ M	10^−6^ M	10^−7^ M
ADSCs	++++	–	–	++	++	+++
UCSCs	++++	–	–	++	++	+++

## Discussion

The ability of ADSCs to differentiate into different types of cells is widely used in tissue engineering and regenerative medicine. Several studies described the use ADSC in tissue remodeling, stimulation of peripheral nerve repair [14], functional recovery in spinal cord damage [15], diabetes treatments [16] and liver injury repair [17]. The ADSCs are also used as filler in plastic and cosmetic surgery [18] and as the autologous chondrocytes alternative for a repair of articular cartilage in the knee [19]. The ADSCs were also used, in combination with fibrin glue and a biodegradable scaffold, to repair the calvarial bone defects [20]. For all these applications the ADSCs should be isolated from healthy and young donors.

Recently, Alt et al. [21] demonstrated that the number of tissue resident ADSCs decrease significantly with the increasing donor age. The ADSCs isolated from old donors display senescent features compared with cells isolated from young donors. Li et al. [22] reported that the donor’s age has the impact on proliferation of human bone marrow MSCs. Similarly, Paxson et al. [23] showed the lower number of colony forming units, lower growth potential and telomerase activity in lung-derived MSCs from older mice. Choudhery et al. [24] reported the effect of donor age on expansion and differentiation potential of human ADSCs and showed that the ADSCs from older donors had reduced viability, proliferation and differentiation potential. In contrast, Legzdina et al. [25] did not found age-related decrease in potential of growth and differentiation of ADSCs isolated from human donors. Authors concluded that the differences between studies from different laboratories resulted from donor-specificity and the intricacy in the dynamics of cell subsets present in ADSC population. In addition it has been shown that various diseases may affect number, proliferation and differentiation properties of MSCs [26].

In presented here study we evaluated how exposure to lipophilic substance HCP affects quality of MSCs (ADSCs and UCSCs). We found three major effects of HCP on MSCs cultured *in vitro*: (1) high toxicity (cell growth arrest) in the highest doses, (2) increased apoptosis especially pronounced in UCSCs, (3) inhibition of differentiation into osteoblasts, which persisted after HCP had been removed before induction of differentiation. The latter is of particular interest because it suggests that HCP causes long-lasting, or even permanent, changes in cell homeostasis, which inhibits the differentiation process. Similar long-lasting effects of the milieu on MSCs’ quality had been reported by others. Kim et al. [27] compared UCSCs isolated from gestational diabetes mellitus (GDM) and healthy pregnant women (N). GDM-UCSCs displayed decreased cell growth and significantly lower osteogenic and adipogenic differentiation potential then N-UCSCs. Furthermore, GDM-UCSCs exhibited earlier cellular senescence, low mitochondrial activity and reduced expression of the mitochondrial regulatory genes. Also Liu et al. [28] reported that MSCs isolated from bone marrow gestational diabetes mellitus patients exhibited lower growth than those isolated from a control group.

Relatively little is known about the biochemical mechanisms of toxicity of HCP at the cellular and molecular level. According to the literature, HCP is a potent hemolytic agent inducing hemolysis by association witch the cellular membrane of red blood cells (RBC). Moreover, residues of HPC were detected in the cytoplasm of surviving RBCs [29]. Amdur et al. [30] showed that HCP binds tightly to cell membrane resulting in the loss of ion gradients. The major effect of HCP is the modification of the permeability of cellular membrane, and in consequence, the disruption of the efflux of monovalent cations such as K^+^ and Na^+^. The ultimate result of this process is osmotic swelling and subsequent hemolysis of RBCs. Osmoregulation of the erythrocytes is based on the balance between the active and passive transport of K^+^ and Na^+^. Hemolysis may be the effect of the disruption of this balance [31]. The defective ion transport can lead to: direct inhibition of (K^+^–Na^+^)-activated and Mg^2+^-dependent ATPase and decreased efficiency of metabolic reactions. These in turn lead to inhibition of oxidation and changes in permeability of the cellular membrane [29]. Toxic mechanism of HCP associated with modifications of cell membrane permeability and mitochondrial function partially explains the results in our present study.

The HCP also influences the cellular energetic processes. It has been shown that HCP in micromolar concentration upregulated respiration of rat liver and calf brain’s mitochondria [32]. In higher concentrations, the HCP acted as a potent uncoupler of oxidative phosphorylation in mitochondria and inhibited mitochondrial respiration. The chemical structure of HCP is similar to that of pentachlorothiophenol and halogenated phenols, which are known as uncoupling agents of mitochondrial oxidative phosphorylation [32]. We observed that 10^−4^ M HCP caused both necrosis and apoptosis of MSCs while the lower concentration (10^−5^ M) of HCP induced apoptosis. It is well known that disruption of mitochondrial functions is a major factor inducing apoptosis [33]. One of the mechanism by which HCP may affect cell apoptosis is its impact on Wnt/β-catenin classic pathway [34]. Park et al. [35] reported that HCP addition to colon cancer cell line inhibited Wnt/-catenin signaling via degradation of the intracellular β-catenin which was independent of GSK-3 and β-TrCP activation. Moreover HCP treatment inhibited proliferation of colon cancer cells. Wnt/β-catenin signaling pathway regulates function of T cells transcription factor (TCF) and affects cellular processes such as embryogenesis, survival, differentiation and proliferation in cells [36]. This may also partly explain our results: attenuated proliferation and differentiation after HCP addition.

We also showed that not only a constant presence of 10^−4^ M HCP in the differentiating medium inhibited MSCs osteogenesis but that also preincubation (for 4–5 days only) of MSCs with the 10^−4^ M HCP inhibited osteogenesis. This suggests that HCP causes permanent changes in metabolism or genetics of MSCs. Because HCP is permanently present in environment and accumulates in human body its harmful effects on MSCs have to be taken into consideration before medical application.
